# Supplementary material for: The interactome of intact mitochondria by cross-linking mass spectrometry provides evidence for coexisting respiratory supercomplexes
Source: Mol Cell Proteomics. 2017 Dec 8;17(2):216–32. doi: 10.1074/mcp.RA117.000470 (PMC5795388; doi:10.1074/mcp.RA117.000470)
Supplement: Supplemental Data [file supp_RA117.000470_133922_0_supp_23978_fzffwf.zip › spectra_annotation/mito_spectra_annotation/472-1-12-1-13-1.pdf]

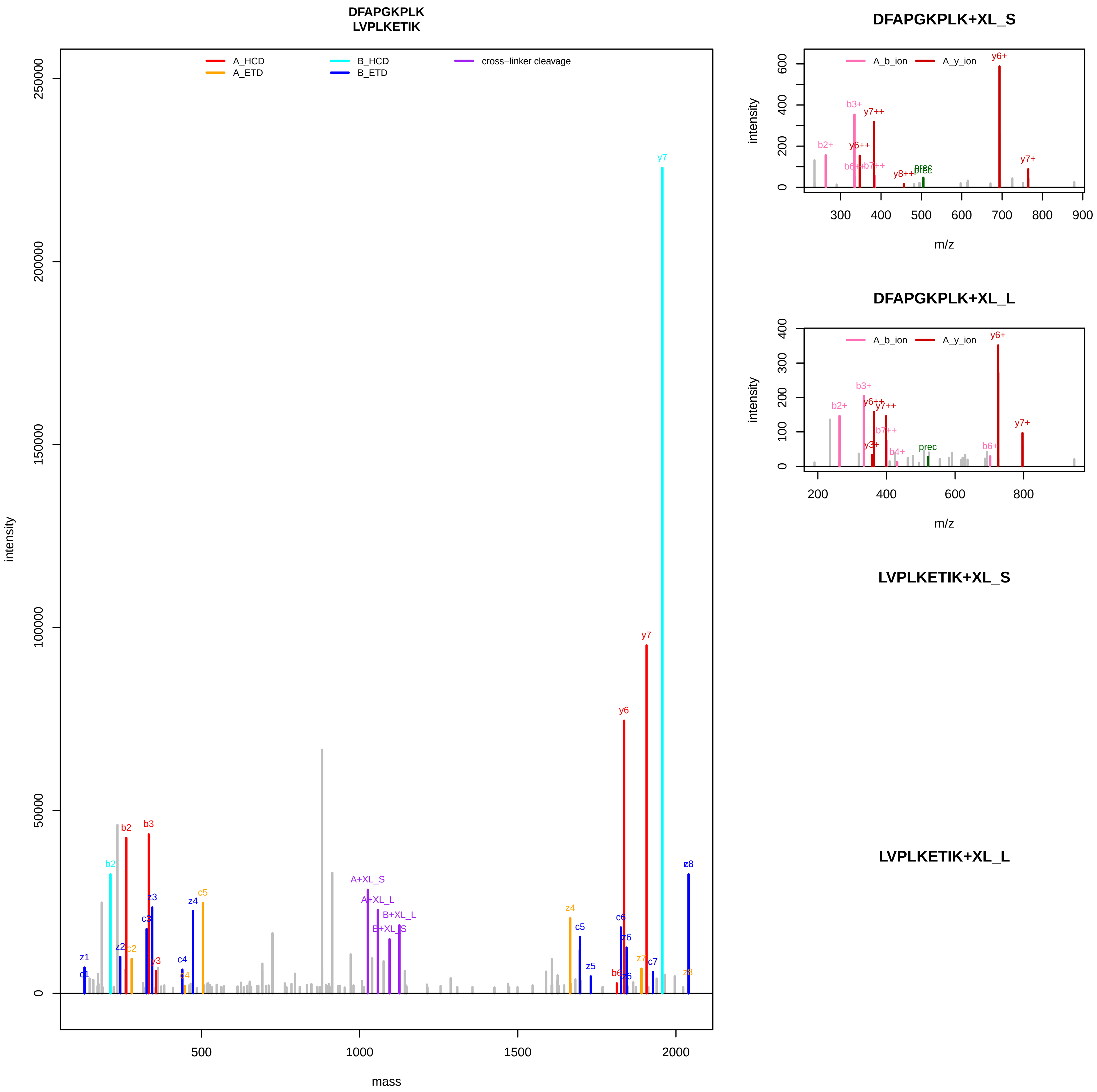
DFAPGKPLK
LVPLKETIK
A_HCD
A_ETD
B_HCD
B_ETD
cross−linker cleavage
intensity
mass
0
50000
100000
150000
200000
250000
500
1000
1500
2000
y7
b2
b3
z3
c3
z2
c2
y3
z1
c1
c4
z4
c5
A+XL_S
A+XL_L
B+XL_L
B+XL_S
z4
c5
z5
c6
z6
b6
y6
y7
z7
c7
z8
DFAPGKPLK+XL_S
A_b_ion
A_y_ion
y6+
b3+
y7++
b2+
y6++
b6++
b7++
y8++
prec
y7+
intensity
m/z
DFAPGKPLK+XL_L
A_b_ion
A_y_ion
y6+
b3+
b2+
y6++
y7++
b7++
y3+
b4+
prec
b6+
y7+
intensity
m/z
LVPLKETIK+XL_S
LVPLKETIK+XL_L
